# A Flight Sensory-Motor to Olfactory Processing Circuit in the Moth *Manduca sexta*

**DOI:** 10.3389/fncir.2016.00005

**Published:** 2016-02-16

**Authors:** Samual P. Bradley, Phillip D. Chapman, Kristyn M. Lizbinski, Kevin C. Daly, Andrew M. Dacks

**Affiliations:** Department of Biology, West Virginia University, MorgantownWV, USA

**Keywords:** modulation, histamine, olfaction, insect, flight

## Abstract

Neural circuits projecting information from motor to sensory pathways are common across sensory domains. These circuits typically modify sensory function as a result of motor pattern activation; this is particularly so in cases where the resultant behavior affects the sensory experience or its processing. However, such circuits have not been observed projecting to an olfactory pathway in any species despite well characterized active sampling behaviors that produce reafferent mechanical stimuli, such as sniffing in mammals and wing beating in the moth *Manduca sexta*. In this study we characterize a circuit that connects a flight sensory-motor center to an olfactory center in *Manduca*. This circuit consists of a single pair of histamine immunoreactive (HA-ir) neurons that project from the mesothoracic ganglion to innervate a subset of ventral antennal lobe (AL) glomeruli. Furthermore, within the AL we show that the *M. sexta* histamine B receptor (MsHisClB) is exclusively expressed by a subset of GABAergic and peptidergic LNs, which broadly project to all olfactory glomeruli. Finally, the HA-ir cell pair is present in fifth stage instar larvae; however, the absence of MsHisClB-ir in the larval antennal center indicates that the circuit is incomplete prior to metamorphosis and importantly prior to the expression of flight behavior. Although the functional consequences of this circuit remain unknown, these results provide the first detailed description of a circuit that interconnects an olfactory system with motor centers driving flight behaviors including odor-guided flight.

## Introduction

Animals exhibit stereotypical search behaviors in pursuit of potential food sources or mating partners. More specifically, some animals employ sampling strategies where rhythmic motor patterns optimize the interaction between stimuli and their affected sensory systems. Consequently, many of these motor systems project to and modulate how sensory systems process this information. For example, saccadic eye movements allow us to focus on objects despite having a fast adapting visual system (Martinez-Conde et al., 2006). Here the neural circuits driving these small movements also send a signal canceling the perception of a moving scene, therefore affording proper behavioral responses to other stimuli in the environment (Zaretsky and Rowell, 1979; Ross et al., 2001). Other motor to sensory circuits have been shown to amplify self-induced communication signals (Mohr et al., 2003), inhibit reflex responses (Chalfie et al., 1985), and are involved in sensory/motor planning (Brainard and Doupe, 2000; Sommer and Wurtz, 2002). While work in other sensory systems have made significant progress in characterizing motor to sensory circuits (Crapse and Sommer, 2008), it is not clear whether such circuits are present in the olfactory system.

When tracking odors, animals typically exhibit behaviors, such as sniffing, that periodically structure olfactory stimuli (Halpern, 1983). Each sniff cycle draws odor-laden air into the nasal cavity during inhalation and forces air out during exhalation, thus imposing a temporal structure on air/olfactory receptor interactions that persists in the absence of odor (Adrian, 1942; Kepecs et al., 2007). In this manner, sniffing couples reafferent mechanical stimuli with odor stimuli resulting in a temporally structured stimulus that improves physiological (Verhagen et al., 2007), and presumably behavioral performance. In the moth *Manduca sexta*, wing beating causes high frequency oscillations in airflow over the antennae in a manner analogous to sniffing (Sane and Jacobson, 2006). These periodic signals have a potentially strong effect on odor-receptor interactions in moths (Loudon et al., 1994; Loudon and Koehl, 2000) and are effectively tracked by antennal and antennal lobe (AL) neurons (Tripathy et al., 2010). This implies that at least part of the temporal structure of encoding neuron activity is driven by time-dependent fluctuations in stimulus concentration (Christensen et al., 1998; Daly et al., 2011), driven by wing-beating. Simulating wing-beating effects on odor exposure by pulsing odor stimuli at wing beat frequencies increases separation of neural ensemble representations for different odors (Houot et al., 2014) and enhances behavioral performance in psychophysical assays of olfactory acuity (Tripathy et al., 2010; Daly et al., 2013). While AL neurons can track pulsed stimuli when the neck connective is intact (Houot et al., 2014), AL neurons are unable to do so when using isolated head preparations (Christensen et al., 1998; Tripathy et al., 2010). This suggests that the AL receives input from flight sensorimotor centers that affects the temporal fidelity with which the AL encodes odors (Christensen et al., 1998; Tripathy et al., 2010). However, relatively little is known about neural circuits connecting flight sensory-motor centers and the AL.

There is limited data describing input from flight sensory-motor centers to the ALs of *Manduca.* This circuit consists of a single pair of histamine (HA) immunoreactive neurons that project from the mesothoracic ganglion (MsG) and bilaterally innervate both ALs and the antennal mechanosensory and motor center (AMMC) (Homberg and Hildebrand, 1991; Homberg, 1994). The purpose of this study was to provide a detailed morphological description of these mesothoracic to deutocerebral histaminergic neurons (MDHns) and to identify candidate post synaptic targets. Using immunohistochemistry, we found that the MDHns ramify in a subset of ventral glomeruli in the AL, the AL isthmus, and the coarse neuropil. A subset of GABAergic local interneurons (LNs) along with one FMRFamide-ir and one allatotropin-ir (ATR-ir) LN express the *Manduca* homolog of the histamine B receptor subtype (MsHisClB) and thus represent candidate postsynaptic targets of the MDHns. Furthermore, although the MDHns are present in larvae and survive metamorphosis there is no expression of the MsHisClB receptor in larval antennal center (LAC) neurons until after pupation has occurred, suggesting the MDHns only affect olfactory processing in adults. The MDHns therefore represent a novel circuit that provides a potential source of information from a flight sensory-motor integration system to the olfactory system.

## Materials and Methods

### Animals

Animals were raised using a standard diet (Bell and Joachim, 1976) and rearing procedures (Tripathy et al., 2010). Adult moths were kept in brown paper bags and placed in an incubator (Percival Scientific Inc.; 166VLC8) where they were exposed to a 16/8 reverse light dark cycle set to 25°C and 75% humidity. Approximately 10 male or female moths aged 3–9 days were used for all experimental groups. For larval studies, stage 5 instar larvae were dissected with trachea removed. Ten larval nervous systems were used for developmental experiments.

### Immunohistochemistry

Immunolabeling was performed as described previously (Dacks et al., 2010) on both sectioned and whole-mount brains depending upon the preparation. For HA immunolabeling, brains were placed in a 4% N-3-dimethylaminopropyl-N’-ethylcarbodiimide (Sigma–Aldrich, 03449) pre-fixative for 3–4 h at 4°C, before being fixed overnight in 4% paraformaldehyde (Electron Microscope Sciences, 15710) in 1% phosphate buffered saline (PBS; Sigma–Aldrich, SLBC5890) at 4°C. For the MsHisClB antibody, brains were placed in 4% paraformaldehyde (Electron Microscopy Sciences, 15710; pH 7.3–7.5) at 4°C overnight. Following fixation, brains were washed in PBS (pH 6.9). For sectioned tissue, adult brains and ganglia were embedded in 5% agarose (Sigma–Aldrich, SLBJ3744V) and sectioned between 50 and 250 μm (depending on the antibody) using a Leica VT 1000S vibrating microtome. The tissue was washed in PBS with 0.5% Triton^TM^-X100 (PBST; Sigma–Aldrich, 110M0009V), blocked for 1 h with 2% IgG-free BSA, J(ackson Laboratory, 001-000-162) and incubated in primary antibody in blocking solution with 5 mM with sodium azide (PBSAT; Fisher Scientific, S2271). Brains were washed and blocked as above, then incubated in secondary antibody (1:1000 Alexa 488, 546, or 633 in PBSAT; Alexa fluor^®^; Lifescience Technologies) overnight at room temperature except for experiments using MsHisClB and/or GABA in which tissue was incubated at 4°C. SYTO 59 (a nuclear label; Invitrogen^TM^; S11341) was used to outline the LAC. Tissue was washed several times in Tris Buffered Saline (TBS; Bio-Rad, 170-6435) and the tissue was incubated in 1:10,000 SYTO 59 in Tris-HCl (Fisher Scientific, BP153 for 60 min before mounting. All tissue was washed in PBST and PBS, then run through an ascending glycerol (Sigma–Aldrich, BCBN3647V) series (40%, 60%, and 80%) and mounted in Vectashield^®^ (Vector laboratories, ZA1222). For whole-mount preparations, tissue was run through an ascending ethanol (Sigma–Aldrich, SHBF6704V) dilution series (30, 50, 70, 95, and 100%) for 10 min each (after the PBS wash), a 1:1 ethanol methyl salicylate solution for 15 min, and finally mounted in 100% methyl salicylate (Fisher Scientific, MFCD00002214). All primary antibody information (including dilutions used, manufacturer, host-species, immunogen and RRID) is included in **Table 1**.

**Table 1:** Primary antibodies used in this study.

Antigen	Immunogen	Manufacturer, host, monoclonal vs. polyclonal	Catalog #	RRID	Dilution used
Histamine	Synthetic HA coupled to succinylated keyhole limpet Hemocyanin with carbodiimide linker	Immunostar, Rabbit, polyclonal	22939	AB_572245	1:500
Bruchpilot	Bruchpilot peptide sequence (1390-1740) from head homogenate	DSHB, Mouse, monoclonal	Nc-82	AB_2314866	1:50
*Manduca sexta* HA B receptor (MsHisClB)	Histamine B receptor peptide sequence (VNPDIELPQLD)	Bethyl Laboratory (custom), Rabbit, polyclonal	N/A	N/A	1:5000
γ-aminobutyric acid (GABA)	Purified GABA conjugated to BSA	Abcam, Mouse, monoclonal	ab49675	AB_880138	1:500
Allatotropin	Allatotropin coupled to thyroglobulin with glutaraldehyde	Dr. Jan Veenstra, Rabbit, polyclonal	N/A	AB_2313973	1:8^∗^
FMRF-amide	Synthetic FMRF coupled to bovine thyroglobulin with gluteraldehyde	Dr. Eve Marder, Rabbit, Polyclonal	N/A	AB_572232	1:8^∗^

*^∗^See fluorescent tagging subsection of the methods for details.*

### Antibody Manufacturing and Characterization

#### Rabbit Anti-Histamine

**FIGURE 1:**
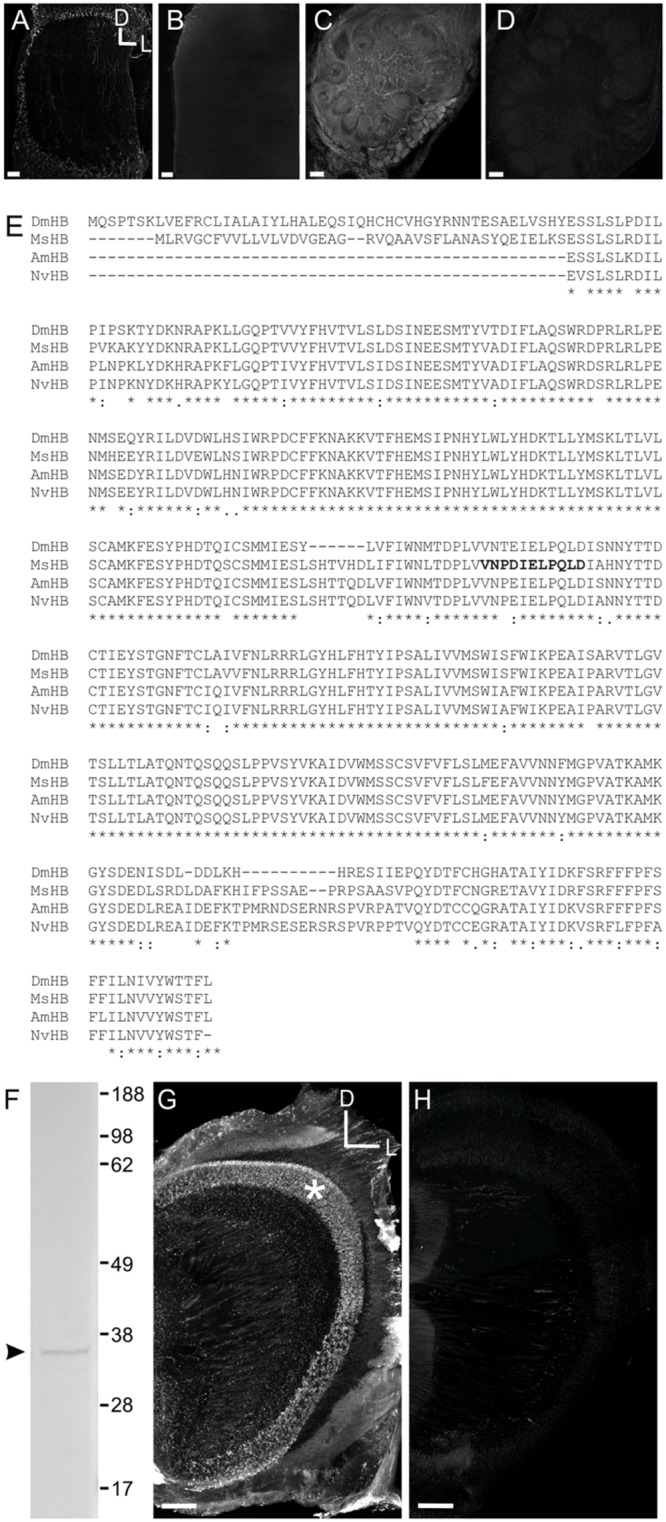
**Characterization of the HA GABA and *Manduca sexta* HA B receptor (MsHisClB) antibodies. (A)** HA labeling in control animals where the antibody was not pre-adsorbed. HA labeling in the optic lobe which is the primary neurotransmitter used by arthropod receptor cells. **(B)** HA labeling in the optic lobe is abolished after the HA antibody was pre-adsorbed with a 10:1 HA to antibody solution. **(C)** GABA labeling remains in control animals where the antibody was not pre-adsorbed with GABA. **(D)** GABA labeling in the AL is abolished after the GABA antibody was pre-adsorbed with a 10:1 GABA to antibody solution. For each panel the same dorsal lateral axis is used. **(E)** Amino acid sequence alignment of the HA B receptor subunits of *Manduca sexta* (MsHB; Msex2.04603-RA), *Drosophila melanogaster* (DmHB; ACA13298.1), *Apis mellifera* (AmHB; ABG75740.1), and *Nasonia vitripennis* (NvHB; ACZ51422.1). Asterisks indicate sequence identity across all four species. Bold font indicates the immunogenic peptide sequence from *Manduca* that was used to generate the MsHisClB antibody. **(F)** Western blot using MsHisClB receptor antibody on *Manduca* brain tissue resulted in a single band at the predicted molecular weight (36 kDa) of the MsHisClB protein. **(G)** Frontal section of optic lobe depicting MsHisClB-ir in the lamina (as labeled by an asterisks). **(H)** Pre-adsorption with the immunogenic peptide sequence eliminates all labeling in the lamina. Scale bars = 50 μm. D, dorsal, L, lateral, A, anterior.

The HA antiserum was raised against synthetic HA conjugated via a carbodiimide linker to succinylated keyhole limpet hemocyanin. Control studies showed that the antibody had no cross reactivity with L-histidine or L-histidine containing peptides, and pre-adsorbing the antiserum with the HA conjugate eliminates labeling (Immunostar histochemical HA antiserum specification sheet) as did an RNAi knock down of histidine decarboxylase in *Drosophila* (Melzig et al., 1996). Finally, pre-adsorbing the HA antiserum against keyhole limpet hemocyanin alone did not eliminate HA labeling in *Bombus impatiens* (Dacks et al., 2010). Pre-adsorption controls in *Manduca* tissue were performed by incubating the rabbit anti-HA antiserum for 24 h in blocking solution (1 mg/ml BSA in PBSAT) with HA (Sigma–Aldrich, H7250) at a ratio of 10:1 HA:antiserum. Non-pre-adsorbed controls in which rabbit anti-HA antibody was incubated in parallel under identical conditions resulted in immunolabeling (**Figure 1A**; *n* = 5) whereas preadsorbing the antibody abolished all staining in *Manduca* optic lobe tissue (**Figure 1B**; *n* = 5).

#### Mouse Anti-Bruchpilot

Bruchpilot (BRP) is homologous to the protein ELKS/CAST in mammals and functions as a structural protein at presynaptic active zones (Wagh et al., 2006). The BRP antiserum was raised against BRP and western blots showed two bands for two isoforms of the BRP protein in *Drosophila* (Wagh et al., 2006). BRP labeling was absent in BRP mutants (Kittel et al., 2006) and has been shown to bind to amino acid sequence 1390–1740 (Fouquet et al., 2009). The BRP antiserum produced a single band at the predicted weight for the *Manduca* homolog of BRP in western blots using *Manduca* brain tissue (Lizbinski et al., 2015). The purpose of using the anti-BRP antibody in this study was to highlight the boundaries of neuropil, rather than to make any conclusions about the distribution of the *Manduca* homolog of BRP.

#### Mouse Anti-GABA

GABA antiserum was raised against GABA coupled to BSA with glutaraldehyde. Controls show that the antibody was highly specific to GABA and did not react with other amino acid BSA conjugates (Abcam data sheet). Pre-adsorption controls were performed by incubating the mouse anti-GABA antiserum for 24 h in blocking solution (1 mg/ml BSA in PBSAT) with GABA (Sigma–Aldrich, cat # A2129) at a ratio of 10:1 GABA:antiserum. Non-pre-adsorbed controls in which mouse anti-GABA antibody was incubated in parallel under identical conditions resulted in strong immunolabeling (**Figure 1C**; *n* = 5) whereas preadsorbing the antibody abolished all staining in *Manduca* AL tissue (**Figure 1D**; *n* = 5).

#### Rabbit Anti-FMRFamide

FMRFamide antiserum was provided by Dr. Eve Marder and was raised against synthetic RF-amide coupled to bovine thyroglobulin with glutaraldehyde (Marder et al., 1987). Preadsorbing the antiserum against synthetic FMRFamide eliminated labeling in larval *Manduca* nervous tissue (Witten and Truman, 1996).

#### Rabbit Anti-Allatotropin

Allatotropin (ATR) antiserum was provided by Dr. Jan Veenstra and raised against purified ATR coupled to thyroglobulin using glutaraldehyde (Veenstra and Hagedorn, 1993). ELISA did not show cross reactivity with 100 pmol corazonin, vasopressin, leucokinin IV, or proctolin, but did show significant immunoreacitivity to the truncated 6–13 analog of *Manduca* ATR (Veenstra and Hagedorn, 1993). Preadsorbing the antiserum against ATR eliminated immunolabeling in *Manduca* tissue (Utz et al., 2008).

#### Rabbit Anti-MsHisClB

To determine the amino acid sequence of the *Manduca* homolog of the HA B-type receptor (MsHisClB), we used the *Manduca* genome (Agricultural Pest Genomics Resource Database^1^: to perform a forward protein BLAST analysis of the *Drosophila melanogaster* histamine B-type receptor (HisClB) amino acid sequence (ACA13298.1). The top match from the *Manduca* genome had an e-value of 0.0 (Msex2.04603-RA). We then reverse blasted this sequence from the *Manduca* genome into the *Drosophila* genome in NCBI and the first three matches were *Drosophila* HisClB isoforms (NP_650116.2, NP_731632.1, and NP_001163591.1), all of which had e-values of 0.0. The next highest match from the *Drosophila* genome was the HisClA receptor (otherwise known as “ora transientless”; NP_524406.1) which is the other of the two HA receptor types in *Drosophila* (Zheng et al., 2002) and had e-values of 3e-148 which is consistent with both HA receptor types having high sequence homology (Zheng et al., 2002; Jones et al., 2010). To ensure that there were not two predicted amino acid sequences from the *Manduca* genome with high sequence homology to the *Drosophila* HisClB receptor, we took the amino acid sequence from the *Manduca* genome with the second highest *e*-value for the *Drosophila* MsHisClB (Msex2.04216-RA; *e*-value = 1e-119) and ran a BLAST analysis of this sequence in the *Drosophila* genome. The BLAST analysis resulted in an e-value of 7.37e-158 for the *Drosophila* ora transientless indicating that the *Manduca* protein with the next closest sequence similarity to *Drosophila* HisClA was likely not the MsHisClB homolog. **Figure 1E** is a sequence alignment of the *Manduca* HisClB receptor (MsHisClB) with the sequences for known histamine B receptors from *Drosophila melanogaster* (ACA13298.1), *Apis meliferia* (ABG75740.1), and *Nasonia vitripennis* (ACZ51422.1) (Jones et al., 2010) using the EMBL-EBI Clustal omega tool^2^ (Sievers et al., 2011).

Custom affinity purified antibodies were generated in rabbit (Bethyl laboratories) using Cys-VNPDIELPQLD as the immunogenic sequence. The immunogenic sequence was highly conserved across *D. melanogaster, A. mellifera*, and *N. vitripennis* (**Figure 1E**). For western blots, adult brains were placed in Bolt^TM^ LDS Sample Buffer (Life Technologies, B0007, Life Technologies) with protease inhibitor cocktail (Research Products International, P50900) and DNase I (Invitrogen, 18068-015) and kept on ice for homogenization with a pestle. Samples were heated in a water bath for 10 min at 95°C. We used the Novex^®^ Bolt^TM^ Gel Electrophoresis System (Life Technologies) with Tris-Glycine SDS Running Buffer at 165V for 2.5 h and Bolt^TM^ 4–12% Bis-Tris Plus Precast Gels (BG04120BOX) to resolve proteins. We used the iBlot^®^ Gel Transfer Device (Life Technologies, IB1001) program P0 (20 V for 1 min, 23 V for 4 min, 25 V for 2 min) to transfer proteins to nitrocellulose membranes (nitrocellulose iBlot^®^ Transfer Stacks, Life Technologies, IB3010-01). The WesternBreeze^®^ Chromogenic Western Blot Immunodetection Kit (WB7105, anti-rabbit) protocol was used to detect proteins. Images of membranes were taken with FluorChem Q using Alpha View Analysis Software. The amino acid sequence of the MsHisClB receptor has a predicted molecular weight of 36 kDa^3^ (ExPASy Bioinformatics Resource Portal) and the western blot resulted in a single band at the predicted molecular weight of 36 kDa (**Figure 1F**). HA is the primary neurotransmitter of arthropod photoreceptors (Hardie, 1989; Stuart, 1999) and the HisClB receptor is expressed by glial cells in the lamina of *Drosophila* (Pantazis et al., 2008). Consistent with this, we observed a band of MsHisClB labeling in the lamina (**Figure 1G**). Pre-adsorbing the MsHisClB antibody in a 10:1 antigenic peptide to antibody solution eliminated all labeling (**Figure 1H**). Pre-adsorption controls were run concurrently with samples incubated in antibody that had not been pre-absorbed with the antigenic peptide (**Figure 1G**), but otherwise treated identically. Scan settings were increased slightly for preadsorbed tissue so that autoflourescence outlined brain structures. Finally, RT-PCR of the insect HisClA showed no band at the predicted height for the receptor (data not shown) suggesting that the MsHisClB receptor is the only HA receptor expressed in AL tissue.

#### Direct Fluorescent Tagging of Primary Antibodies

Both neuropeptide antibodies (anti-FMRFamide and anti-ATR) and the MsHisClB receptor antibody were produced in rabbit hosts. Therefore, to double label using the neuropeptides and the rabbit anti-MsHisClB antibodies we directly fluorescently tagged each primary antibody using the APEX antibody labeling kit (Life Technologies, A10468 488, A10475 for 647; Woo et al., 2010). This method covalently bonds the IgG antibody to a fluorescent label, and therefore eliminates cross reactivity of secondary antibodies with primary antibodies raised in the same animal. To remove contaminants, the labeling tip was hydrated with 100 μL of wash buffer to which 10–20 μg of IgG antibody is added and eluted with a syringe: 10 μL of MsHisClB, and 1 μL of both FMRFamide and ATR antibody, respectively. This solution was then combined with reactive dye (either Alexa 488 or Alexa 647) containing 2 μL of DMSO and 18 μL of labeling buffer. This solution then incubated for 2 h at room temperature. The solution was washed with 50 μL of buffer and eluted through the tip. Finally, 40 μL of elution buffer is eluted through the tip and mixed with 10 μL of neutralization buffer to yield a final volume of ∼50 μL of solution. This solution was then diluted in 350 μL of PBSAT and tissue was incubated for 48 h at 4°C.

#### Retrograde Dye Fills of AL PN Output Tracks

Two to 3 days-old-moths were restrained with dental wax and the head capsule was opened. Once opened, dextran-Texas Red dye (ThermoFisher, D-1863) was injected into either the mushroom bodies or lateral horn (the two projection fields of AL PNs). Animals were kept alive for 2–3 days post injection and were fed sugar water to ensure that they survived. After 2–3 days, animals were sacrificed and ran through the HA staining protocol described above.

### Ablation Studies

**FIGURE 2:**
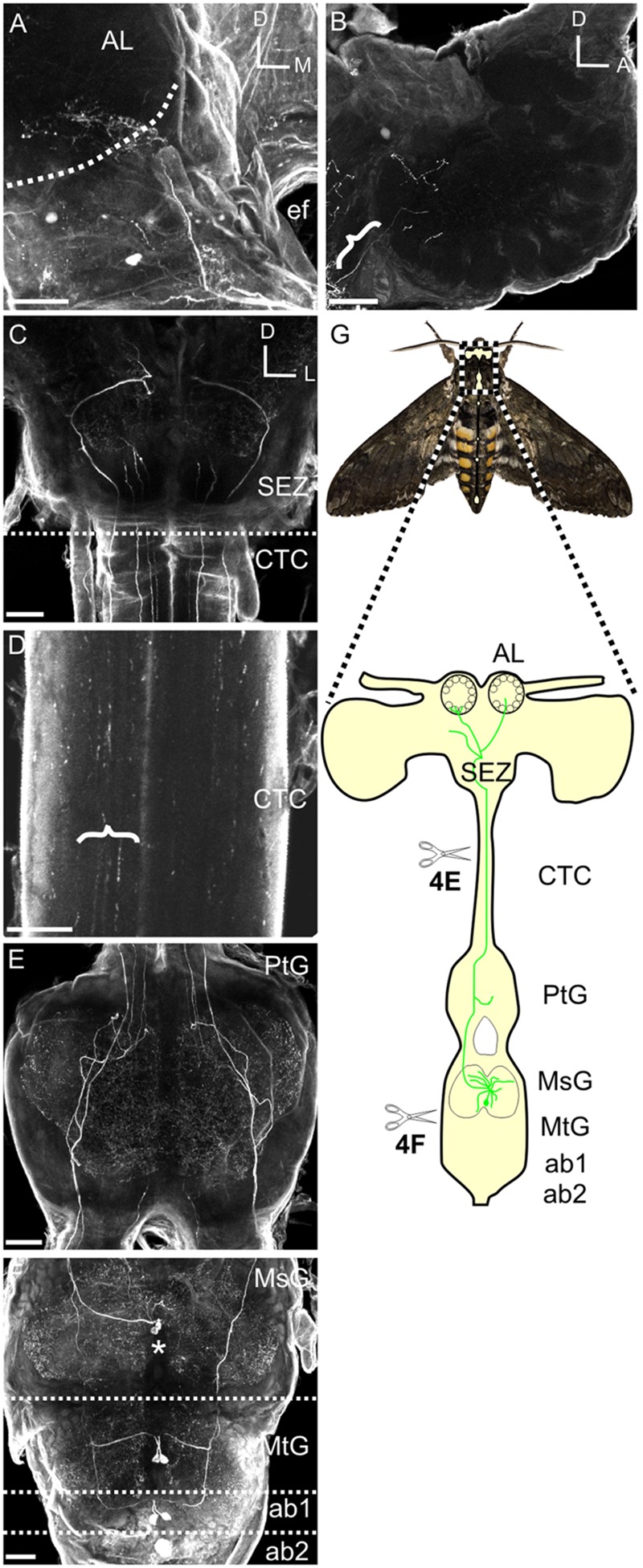
**Mesothoracic deutocerebrum histamine neurons project from the MsG to the AL of *Manduca sexta*. (A)** Frontal view of HA-ir labeling in a whole mount brain preparation. Hatched line outlines the AL boundary. **(B)** Saggital view of a HA-ir process entering the AL (bracket). **(C)** Frontal view of HA-ir processes entering the SEZ from the cervicothoracic connective. Notice that four pairs enter the SEZ. **(D)** HA-ir processes in the cervicothoracic connective. Brackets highlight three HA-ir processes. **(E)** Horizontal view of the HA-ir processes in the prothoracic ganglion. Notice four pairs ascending from here as well. **(F)** Horizontonal view of HA-ir in the MsG, the metathoracic ganglion, and the first two abdominal ganglia. Each SEZment has a pair of HA-ir cell bodies located in the medial third of their respective ganglion. Asterisks highlights MDNn cell bodies. **(G)** Schematic of the *Manduca* nervous system highlighting the MDHns (green). Hatched boundary indicates the MsG. All scale bars = 100 μm. AL, antennal lobe; ef, esophageal foramen; SEZ, subesophageal zone; CTC, cervicothoracic connective; PtG, prothoracic ganglion; MsG, mesothoracic ganglion; MtG, metathoracic ganglion; ab1, abdominal ganglion 1; ab2, abdominal ganglion 2.

To definitively demonstrate that the MDHns are the sole source of HA to the AL, lesion experiments were performed to ablate ascending HA-ir fibers from the MDHns or more posterior HA-ir neurons in the metathoracic and abdominal ganglia. At 1–3 days post-eclosion the connective between the subesophageal zone (SEZ) and the prothoracic ganglion was lesioned to destroy all ascending input to the brain from the thoracic and abdominal ganglia (including the MDHns; see dashed line in **Figure 2D**) or the divide between the mesothoracic and metathoracic ganglia was cut to destroy all ascending cells posterior to the MsG, (including pairs of HA cells in the metathoracic ganglia and the first two abdominal ganglia; see dashed line between the MsG and the MtG in **Figure 2F**). Moths were fed sugar water each day following the ablation to increase survival rates. After 8 days, the brains were dissected for immunolabeling for HA-ir and BRP-ir. For the ablation of the connective between the prothoracic ganglion and SEZ we used 6 moths in which we cut the connective between the prothoracic ganglion and the SEZ and 6 sham operated moths. Successful ablation was verified by a lack of HA-ir in the remnants of the connective, while sham ablation (when the connective was not cut) was verified by the presence of HA-ir in the remnants of the connective. For the ablation of the boundary between the mesothoracic and metathoracic ganglia, successful ablation was verified by a lack of HA-ir fibers in the MsG that originate from the more posterior ganglia. In 10 moths, 2 moths resulted in the successful elimination of the ascending fibers from the metathoracic ganglion, but this did not result in loss of HA-ir in the AL.

### Confocal Microscopy

Optical stacks were acquired using an Olympus Fluoview FV 1000 confocal microscope. All scans were taken with either a 20X or 40X oil lens. Confocal planes were stacked with optimized step sizes for the given objective (1.79 μm for 20X and 0.54 μm for 40X) in the Fluoview viewer software (FV10-ASW Version 04.00.02.09). All images were scanned at either 512 × 512 or 1024 × 1024 pixel resolution. Cell body counts and size measurements were performed in Fluoview. Corel Draw (Version 13.0.0.576) was used to organize figures. Vaa3D (Peng et al., 2010) was used to generate 3D reconstructions of confocal stacks that could be rotated to resolve the degree to which structures physically overlap.

## Results

### Two HA Immunoreactive Cells Project from the MsG to the AL

Although motor-to-sensory circuits have been extensively characterized in many sensory systems, there is a dearth of detailed descriptions of input from motor to olfactory centers. The purpose of this study was to extensively characterize the structure, candidate targets and development of a motor-to-olfactory circuit. In *Manduca* a pair of HA-ir cells (the MDHns) project from the MsG to the AL (Homberg, 1994). However, there is very little known about the fine morphological details of MDHns in either the MsG or the AL. Furthermore, nothing is known about the potential targets of the MDHns or their development through metamorphosis. **Figure 2** shows the MDHns in the nervous system including the brain (**Figure 2A**), entering the AL (**Figure 2B**), entering the SEZ from the neck connective (**Figure 2C**), in the neck connective (**Figure 2D**), in the prothoracic ganglion (**Figure 2E**), and in the MsG (**Figure 2F**; *n* = 54).

**FIGURE 3:**
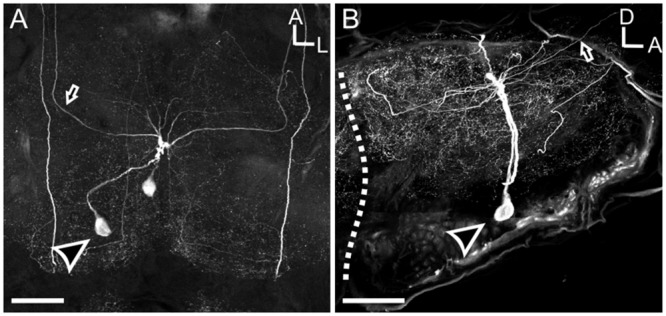
**Mesothoracic deutocerebrum histamine neurons processes radiate laterally throughout the MsG, but are primarily restricted to the dorsal aspect. (A)** Horizontal view of the MSG showing two cell bodies with each cell projecting out one side of the ganglia. **(B)** Sagittal section of the MsG shows two large HA-ir cells with cell bodies (black arrow head with a white outline) situated ventrally and a radiating dendritic field dorsally with the axon (black arrow with white outline) projecting up the connective between the mesothoracic and prothoracic ganglia. White dotted line indicates the boundary between the mesothoracic and metathoracic ganglia. Arrow indicates MDHn cell body in each image. All scale bars = 100 μm.

**FIGURE 4:**
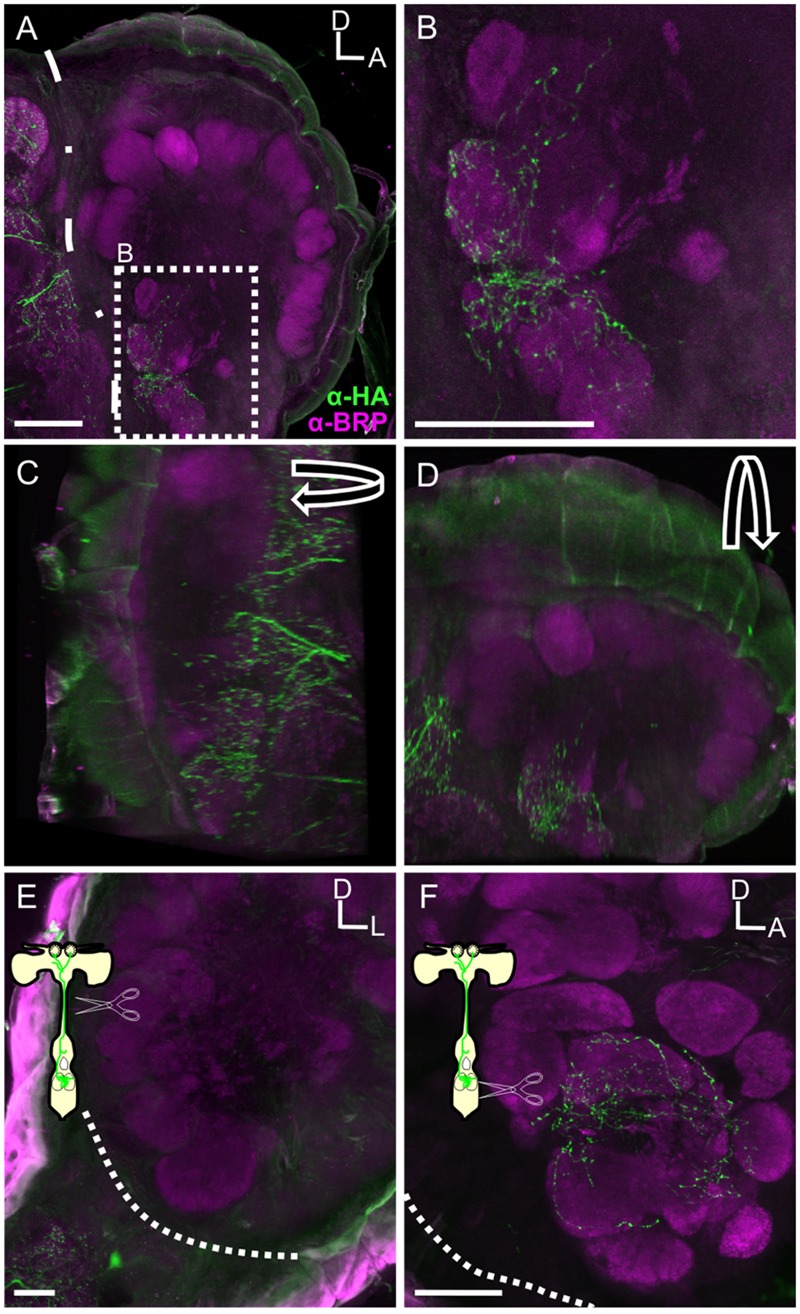
**The MDH neurons provide the sole source of HA-ir input to the ALs. (A)** Saggital section of the AL with HA-ir (green). BRP (magenta) outlines glomeruli of the AL. Dotted line outlines the posterior boundary of the AL. Scale bar = 100 μm. **(B)** High magnification view of inset from **(A)**. Highly varicose HA-ir processes innervate 4–6 ventral posterior glomeruli. Scale bar = 50 μm. **(C)** Rotation of image **(A)** about the *y*-axis showing HA still overlapping with BRP labeling. **(D)** Rotation of image **(A)** about the *x*-axis again showing HA overlapping with BRP labeling, collectively confirming that HA ramifies glomeruli. **(E)** Frontal section showing that HA-ir is absent in the AL following ablation of the cervicothoracic connective. Scale bar = 100 μm. **(F)** Sagittal view of HA-ir in the AL following ablation between the MsG and the metathoracic ganglia in which the lesioning of metathoric HA-ir neuron axons was confirmed. Dashed lines indicate boundary of AL in **(E,F)**. Scale bars = 50 μm.

The large MDHn cell bodies (∼60 μm in diameter) are located on the ventral surface of the MsG (**Figure 3A**) near the intersection of the sagittal and coronal midlines, and extend large primary neurites to the dorsal MsG (**Figure 3A**; *n* = 30). In the dorsal MsG, the MDHns produce a radial planar sheet of processes, with occasional sparse innervation of the ventral MsG (**Figure 3B**). Each MDHn extends a single axon ipsilaterally through the prothoracic ganglion and SEZ (**Figures 2E** and **3A,B**), and bilaterally arborizes in the ventral AL (**Figures 2A** and **4A**). To determine the extent to which the MDHns innervate the AL, we used the BRP antibody to delineate glomerular boundaries and immuno-labeled for HA. Varicose HA-ir processes extensively innervate a subset of ventral posterior glomeruli (**Figures 4A,B**; *n* = 21) and extend sparsely into the ventral posterior coarse neuropil of the AL. Reconstructing and rotating the confocal image stack confirms that the HA-ir processes both encapsulate and innervate the glomeruli (**Figures 4C,D**). There is not much known about the ventral glomeruli in *Manduca* other than CO2 being processed in the labial pit organ glomerulus (Guerenstein et al., 2004), therefore why the MDHns are restricted to this area of AL is unclear.

In addition to the MDHns, HA-ir neurons in the metathoracic and first abdominal ganglia (**Figure 2F**) extend processes to the brain via the cervicothoracic connectives. The processes of these HA-ir from other ganglia intertwine with those from the MDHn in the prothoracic ganglia (**Figure 2E**), making it difficult to definitively ascribe the HA-ir processes in the AL as belonging exclusively to the MDHns. Furthermore, there are ∼20 pairs of HA-ir neurons in the SEZ and protocerebrum of *Manduca* (Homberg and Hildebrand, 1991). To demonstrate that the HA-ir processes in the AL originate from the MDHns, we performed two ablation experiments (**Figures 4E,F**). In the first experiment, we cut the cervicothoracic connective between the prothoracic ganglion and brain in adult moths and kept the moths alive for 8 days. This protocol eliminates HA-ir processes arising from cells in the thoracic and abdominal ganglia (including the MDHns), but leaves the processes from other HA-ir neurons in the brain intact (notice HA-ir ventral to the AL outlined by dotted line with no HA-ir overlapping with BRP-ir outlining glomeruli **Figure 4E**). Ablation of thoracic and abdominal sources of HA-ir was confirmed via elimination of HA-ir entering the ventral SEZ. Ablating the cervicothoracic connective eliminates all HA-ir in the AL (**Figure 4E**) indicating that the HA-ir processes in the AL originate from the ventral nerve cord, posterior to the cut site. It is possible that cutting the cervicothoracic connectives indirectly affects other HA-ir neurons in the brain, which might contribute to AL HA-ir processes we observe. However, we find no evidence to support this notion. In the second ablation experiment, we lesioned the thoracic ganglia at the boundary between the metathoracic ganglion and MsG. This ablates all ascending HA-ir processes posterior to the MDHns (i.e., the HA-ir cells in the metathoracic and abdominal ganglia) but leaves MDHn processes intact. These experiments show that after ablating the cells posterior to the MDHns that there is still HA-ir in the AL (**Figure 4F**). Together these experiments suggest that the MDHns are the exclusive source of the HA-ir processes in the AL.

### The MsHisClB Receptor is Expressed in a Subset of GABAergic LNs, One FMRFaminergic LN and One Allatotropinergic LN

To determine the candidate targets of the MDHns, antibodies were generated against the *Manduca* homolog of the HA B-type receptor (MsHisClB; **Figure 1** and see Materials and Methods). Insects possess two HA receptor types, HisClA and HisClB (Gisselmann et al., 2002; Zheng et al., 2002), both of which are ligand-gated chloride channels (McClintock and Ache, 1989; Hardie, 1989). Each receptor is homomeric with two genes coding for the two subunits HisCl-α1 and HisCl-α2 (Gisselmann et al., 2002). These receptors are members of the large cys-bridge superfamily of ligand-gated ion channels comprised of four transmembrane domains (Gisselmann et al., 2002). The MsHisCIB antibody produces extensive labeling in the lamina of the optic lobes of *Manduca* where histaminergic photoreceptors terminate (**Figure 1G**) which is consistent with HisClB receptor expression by glial cells in the lamina of *Drosophila* (Pantazis et al., 2008). Within the AL, MsHisClB-ir was observed in every glomerulus, which was surprising as the MDH neurons only innervate a set of ventral glomeruli. The MsHisClB antibody produces only a single band in western blots at the predicted height for the MsHisClB receptor (**Figure 1F**; *n* = 5) and all labeling is eliminated by pre-adsorption with the immunogenic sequence (**Figures 1G,H**; *n* = 6), making it unlikely that this antibody is labeling additional proteins. It is, however, possible that the MsHisClB-ir reflects distribution of the MsHisClB receptor during transport throughout the cell as opposed to distribution of the receptor at functional synapses.

**FIGURE 5:**
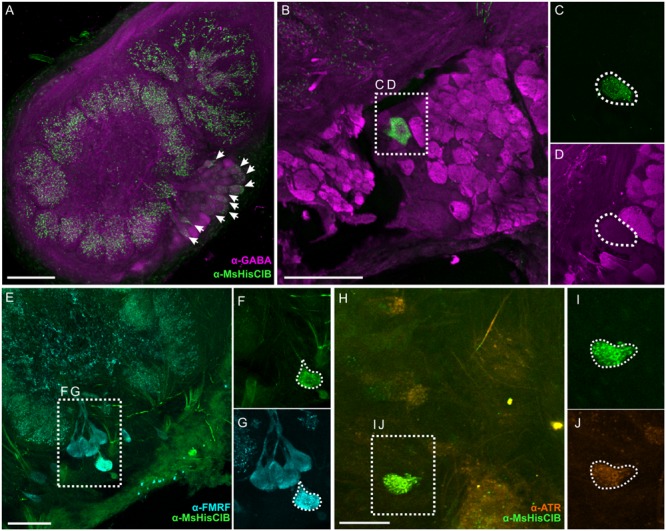
**Within the AL the MsHisClB receptor is expressed by a subset of GABAergic LNs and one FMRF-amidergic and one ATRergic LN. (A)** MsHisClB (green) and GABA (magenta) co-labeling in the lateral cell cluster of the AL. MsHisClB-ir is expressed in all AL glomeruli. Scale bar 100 μm. **(B)** GABA-ir and MsHisClB-ir expression in the lateral cell cluster. **(C,D)** Inset from **(B)** highlights a single large MsHisClB-ir cell body that does not express GABA. **(E)** FMRFamide-ir (cyan) and MsHisClB-ir (green) expression in the lateral cell cluster. **(F,G)** Inset from **(E)** highlights a single large cell body that expresses both MsHisClB-ir and FMRFamide-ir. **(H)** ATR-ir (orange) and MsHisClB-ir (green) expression in the lateral cell cluster. **(I,J)** Inset from **(H)** highlights a single large cell body that expresses both MsHisClB-ir and FMRFamide-ir. All scale bars = 50 μm unless otherwise noted.

In the AL we observed 11 (±0.81 SEM, from 3 moths) and 9.3 (±0.43 SEM, from 3 moths) MsHisClB-ir cell bodies in males and females, respectively, in the lateral cell cluster (**Figure 5A**). The sex differences observed may be due to neurons that project to the macroglomerular complex in males, as we see widespread labeling therein (**Figure 5A**). We observed two classes of MsHisClB labeled cells differing in cell body size. In each AL there were 1–2 larger MsHisClB-ir cells (23.98 μm ± 0.73 SEM diameter; *n* = 10) with the remainder having smaller cell bodies (14.79 μm ± 0.52 SEM diameter; *n* = 10). LN cell bodies are found only in the lateral cell cluster and fall within in two populations based on cell body size being either ∼12 μms or ∼20 μms in diameter (Hoskins et al., 1986) whereas we calculate an average PN cell body size of 8.16 μm (±0.16 SEM) from our retrogradely filled PNs, thus the size of MsHisClB-ir cell bodies is consistent with LNs. Furthermore, we do not observe HA-ir processes innervating any of the AL output tracts (Supplementary Figures S1A,B), nor is there any MsHisClB-ir within the output tracts (Supplementary Figures S1C,D). The MsHisClB-ir neurons collectively branch in every glomerulus (**Figure 5A**; *n* = 37), again consistent with the MsHisClB receptor being expressed by LNs, rather than PNs. To further functionally characterize these MsHisClB-ir cells, we co-labeled for several transmitters, including GABA (Hoskins et al., 1986), FMRFamide (Homberg et al., 1990), and ATR. All but one MsHisClB-ir labeled neuron was GABA-ir (**Figures 5B–D**; *n* = 19) with one cell co-labeled for MsHisClB and FMRFamide and one cell co-labeled for MsHisClB and ATR (**Figures 5E–J**, respectively; *n* = 5,10, respectively). Together these results suggest that any influence of the MDHns on AL processing is exerted via a population of GABAergic and peptidergic LNs. The expression of the MsHisClB receptor by AL neurons and the MDHn being the sole source of HA-ir in the AL suggests that the MDH neurons provide some form of input to the AL. This does not, however, imply that the MDH neurons do not also provide input to circuitry within the MsG. MsHisClB receptor is also expressed within the MsG (Supplementary Figure S2), however, both the MDHns and HA-ir neurons from the metathoracic and abdominal ganglion (**Figure 2F**) innervate the MsG, suggesting that HA also plays a role in network function within the MsG.

### MDHns Survive Metamorphosis but the LAC lacks MsHisClB Expression

**FIGURE 6:**
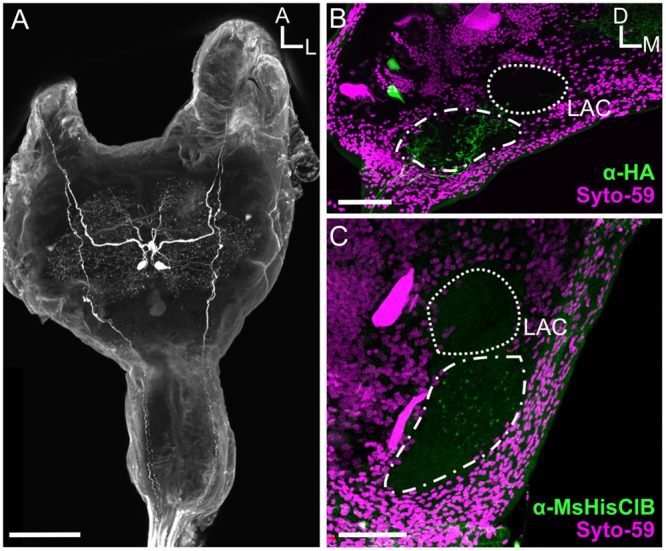
**The MDHns survive metamorphosis, but the MsHisClB receptor is not expressed in the LAC. (A)** Horizontal view of HA-ir in the fifth instar larval MsG shows highly similar cell morphology and radiation patterns of fine processes as in the adult MsG. **(B)** HA-ir in the larval brain (green) shows extensive branching in the tritocerebrum (dash-dot line), but very little innervation in the LAC (dashed line). Syto-59 (magenta) highlights the boundary of the tritocerebrum and LAC. **(C)** MsHisClB-ir (green) is present in the tritocerebrum, but not in the LAC. LAC and tritocerebrum highlighted with Syto-59 (magenta) as in **(B)**. All scale bars = 100 μm.

There are many neurons that survive metamorphosis, often being repurposed to take on new tasks to match the dramatic changes in behavioral demands between the larval and adult life stage. In *Manduca*, motor neurons survive metamorphosis, but their morphology and biophysical properties are altered dramatically to allow them to take on life-stage specific tasks, for instance, transitioning from participating in walking motor programs as larvae to flying motor programs as adults (Duch and Levine, 2000). Given that odor-guided flight is an adult specific behavior, we predicted that the MDHns would either not be present or the MsHisClB-ir would not be expressed in the LAC. Similar to adults (see **Figure 3A**), fifth instar larvae have a pair of large HA-ir cells in the MsG that ascend to the brain (**Figure 6A**). As in adults, the cell bodies are also located ventrally near the intersection of the sagittal and horizontal midlines of the MsG, with a single axon ipsilaterally projecting up each connective. Furthermore, the HA-ir processes also radiate in all directions in the dorsal MsG as in the adult. Because the LAC does not express BRP-ir, we used Syto-59 to label the nuclei of cell bodies that surround the LAC (**Figures 6B,C**) as a means of highlighting the boundaries of this brain region. In the larval brain, HA-ir is most abundant in the tritocerebrum (**Figure 6B**; dash line) just ventral and lateral to the larval LAC (small dotted line) with a small amount of HA-ir entering the LAC (*n* = 17). This suggests that the MDHns are present and project to the olfactory system of larval *Manduca*. However, there are no MsHisClB-ir neurons within the LAC, despite the presence of MsHisClB-ir collaterals in the tritocerebrum (**Figure 6C**; *n* = 6). This suggests that while the MDHns provide sparse innervation of the LAC, they likely do not play a functional role in the larval olfactory system, at least via the MsHisClB receptor, although it is possible that the MsHisClA receptor is expressed there. What role this circuit would play in the larval olfactory system is not clear as the larva do not fly, but there could be information pertaining to walking patterns.

## Discussion

**FIGURE 7:**
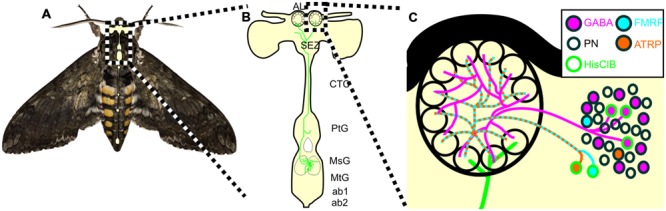
**Schematic of the proposed MDHn circuit. (A)** *Manduca* with overlaid nervous system cartoon. **(B)** Schematic of the MDHn cells from the thoracic ganglia to the AL. Only one cell is shown in detail with processes radiating in the MsG, a small process in the prothoracic ganglion, projecting up the cervicothoracic connective, a branch to the AMMC, and bilateral projections to each AL. **(C)** MDHn projection entering the ventral AL (green) along with the proposed AL circuitry. For the sake of simplicity, only the processes from MsHisClB-ir expressing neurons (green outline) are shown. MsHisClB-ir GABAergic (pink with green outline) and peptidergic (cyan or orange with green outline for FMRFamide and ATR, respectively) LNs ramify each glomerulus. Other cell types are also present including PNs (open circles), GABAergic LNs (pink circles with black outlines), ATR LNs (orange circles with black outline), and FMRF LNs (blue circles with black outline). AL, antennal lobe; oe, esophageal foramen; SEZ, subesophageal zone; CTC, cervicothoracic connective; PtG, prothoracic ganglion; MsG, mesothoracic ganglion; MtG, metathoracic ganglion; ab1, abdominal ganglion 1; ab2, abdominal ganglion 2.

Animals use a variety of behavioral strategies to optimize internal representations of the external world, including repetitive motor patterns that alter stimulus structure. Nervous systems have concurrently evolved circuits that provide information to sensory systems about impending behaviors that will affect sensory input. Although this has been well-documented in many sensory systems, very little is known about neural circuits projecting from neural centers governing odor-guided behaviors to olfactory networks. The goal of this study was to characterize a novel sensory-motor to olfactory circuit that projects from flight sensory-motor centers to the primary olfactory processing center in insects. We found that the MDH circuit provides the only source of HA to the AL and affects a small but diverse population of widely projecting LNs in adult *Manduca* (**Figure 7**). Our data suggest that the MDHns provide histaminergic inhibitory input to the AL that could modify olfactory processing within the context of flight or other MsG mediated activity such as walking patterns.

The MDHn processes project laterally across the MsG (**Figure 3A**), yet are most dense in the dorsal MsG (**Figure 3B**), suggesting that while they may integrate information from both sides of the animal, they are likely to interact with cells that are restricted to the dorsal aspect of the MsG. The MsG contains wing and leg motor neurons, sensory afferents, CPG components, and modulatory neurons some of which occupy specific MsG regions. The dendritic fields of wing elevator and depressor motor neurons are located in the dorsal region of the MsG in *Manduca* (Rind, 1983) whereas most of the sensory afferents from the wings are localized in both the dorsal and ventral MsG in a closely related species of hawkmoth, *Agrius convolvuli* (Ando et al., 2011). In addition, there are a population of non-spiking, GABAergic LNs that project to the dorsal side of the MsG of the locust (Watson and Burrows, 1987), and populations of octopaminergic (Stevenson et al., 1992), serotonergic and dopaminergic neurons (Claassen and Kammer, 1986) that project throughout the MsG. The extensive branching of the MDHns in the MsG suggests that these neurons interact with one or more components of the MsG. The potential cumulative effect of multiple inputs onto MDHns makes understanding the input to this neural circuit challenging. Single neurons releasing multiple neurotransmitters alone can have state dependent effects on network output (Swensen and Marder, 2000; Nusbaum et al., 2001). Furthermore, this complexity is compounded when considering the MDHns impact a heterogeneous population of AL LNs.

Arthropod HA receptors are ligand gated Cl^-^ channels (McClintock and Ache, 1989; Hardie, 1989) sharing ∼45% amino acid similarity to the alpha3 subunit of the human glycine receptor (Zheng et al., 2002), thus the effect of HA on MsHisClB expressing LNs is likely inhibitory in nature. Within the AL there are ∼300 LNs that belong to a diverse set of subtypes based on morphology, neurotransmitter content and physiological response properties (Chou et al., 2010; Reisenman et al., 2011). These LNs mediate diverse processing mechanisms such as lateral inhibition for gain control (Olsen and Wilson, 2008). In addition, these widely branching LNs activate metabotropic receptors whose effects occur on longer and more variable time scales than ionotropic receptors. Therefore the overall network effect of MDHn activity is variable in both the spatial and temporal domain making this circuit difficult to characterize. One potential mechanism would be suppression of GABA, FMRFamide and ATR release by select LNs within the AL. Theoretically, decreasing the influence of these predominantly inhibitory LNs could act to disinhibit the inhibitory AL local network, which could lead to a refinement of PN activity. While the role this refinement has on AL output activity is not clear, it could be in response to the rapid oscillatory nature of the stimulus experience which is driven in part by wing-beating (Sane and Jacobson, 2006). Finally, while invertebrate sensory-motor to sensory circuits typically function to filter reafferent stimuli, we suggest that it is unlikely that the MDHns function in this manner because non-olfactory responses persist in fully intact preparations (Tripathy et al., 2010). Therefore, it may be that MDHn activity indirectly refines PN spatiotemporal response patterns to modify the information output to higher order processing centers during flight. Indeed evidence suggests that the fine temporal structure of AL/OB output patterns contain substantial information about odor identity (Daly et al., 2004; Staudacher et al., 2009; Rebello et al., 2014). However, future studies investigating both the activity patterns of MDHns during flight behavior and the consequences of HA release on AL response properties are necessary to confirm this hypothesis.

Many active sampling behaviors rapidly sample the sensory field providing discrete epochs of input to a sensory system; for example, micro-saccadic eye movements mentioned above. In addition, the details of temporally structured reafference may be dependent on the behavior of the animal. For instance, when exposed to a novel stimulus mice and rats increase their sniff frequencies (Kepecs et al., 2007; Wesson et al., 2008a,b) and sniff frequency modulation is dependent on the specifics of the behavioral task such as free exploration, detection, and discrimination. Insects also show stereotyped active sampling behaviors that are temporally structured. *Bombyx mori* require wing beating to track pheromone plumes despite their inability to fly (Obara, 1979) and male oriental fruit moths continue to fan their wings as they track a calling female even though their final approach is on foot (Baker and Carde, 1979).

From a whole nervous system perspective, it is perhaps not surprising that network-specific processing of information must be adjusted based on inputs from many disparate networks. It is becoming increasingly apparent that networks receive input from a large number of different sources and thus must integrate a variety of ongoing contexts. The mammalian Raphe nuclei provide widespread serotonergic input, yet they also receive input from many other brain areas (Dorocic et al., 2014; Liu et al., 2014; Weissbourd et al., 2014). More specifically, the olfactory systems of animals receive a variety of inputs from other brain regions including serotonergic (Kent et al., 1987; McLean and Shipley, 1987; Dacks et al., 2006), dopaminergic (Dacks et al., 2012), cholinergic (Macrides et al., 1981; Mandairon et al., 2006), octopaminergic (Dacks et al., 2005; Sinakevitch et al., 2005; Sinakevitch and Strausfeld, 2006; Dacks and Nighorn, 2011), and GABAergic (Gracia-Llanes et al., 2010; Nunez-Parra et al., 2013) cells all of which modify sensory processing within different, sometimes competing contexts. Our data support the hypothesis that olfactory processing in *Manduca* may also be adjusted within the context of ongoing activity in the MsG via the histaminergic MDHns.

## Author Contributions

SB, PC, KL, KD, and AD designed research. SB, PC, and KL performed research. SB, PC, KL, KD, and AD analyzed the data. SB, KD, and AD wrote the paper.

## Conflict of Interest Statement

The authors declare that the research was conducted in the absence of any commercial or financial relationships that could be construed as a potential conflict of interest.

## Footnotes

1 www.agripestbase.org

2 http://www.ebi.ac.uk/Tools/msa/clustalo/

3 http://web.expasy.org/compute_pi/

## Abbreviations

AL: antennal lobe
AMMC: antennal mechanosensory and motor center
ATR: allatotropin
BRP: bruchpilot
BSA: bovine serum albumin
FMRF: FMRF-amide
HA: histamine
HisClA: histamine A receptor
LAC: larval antennal center
LNs: local interneurons
MDHn: mesothoracic deutocerebrum histamine neurons
MsHisClB: *Manduca sexta* histamine B receptor
MsG: mesothoracic ganglia
ORNs: olfactory receptor neurons
PNs: projection neurons
SEZ: subesophageal zone
