# Supplementary material for: A Flight Sensory-Motor to Olfactory Processing Circuit in the Moth Manduca sexta
Source: Front Neural Circuits. 2016 Feb 16;10:5. doi: 10.3389/fncir.2016.00005 (PMC4754697; doi:10.3389/fncir.2016.00005)
Supplement: Supplementary file 1 [file Data_Sheet_1.DOCX]

Supplementary Material

**A flight sensory-motor to olfactory processing circuit in the moth *Manduca sexta***

Samual P. Bradley*, Phillip D. Chapman, Kristyn M. Lizbinski, Kevin C. Daly^#^, Andrew M. Dacks^#^

^#^ Denotes co-senior authorship

*** Correspondence:** Samual P. Bradley, sbradle7@mix.wvu.edu.

Supplemental Figure 1. The AL output tracts lack HA-ir and MsHisClB-ir. (A) Horizontal section through the AL output tracts (highlighted via Texas Red PN dye fills from mushroom bodies and lateral horn; magenta) that have been immunolabeled for histamine (green). Scale bar=100 um. (B) Rotation of panel (A) showing that HA-ir does not innervate the AL output tracts. (C) MsHisClB-ir in a horizontal section through the AL output tract (bracket). No MsHisClB-ir was observed within the AL output tracts. Scale bar= 50 um. (D) Inset from panel (C).

Supplemental Figure 2. MsHisClB-ir in the MsG. Two large cell bodies on each side of the ganglion express the receptor and are indicated by arrow heads. Scale bar =50 um.
